# The effectiveness and safety of one-stage iStent-based micro-invasive glaucoma surgery—A retrospective study

**DOI:** 10.3389/fmed.2023.1273889

**Published:** 2023-11-23

**Authors:** Marta Hajduga-Szewczyk, Adrian Smedowski, Iwona Filipecka, Ewa Mrukwa-Kominek

**Affiliations:** ^1^Department of Ophthalmology, Professor K. Gibinski University Clinical Center, Medical University of Silesia, Katowice, Poland; ^2^Okulus Plus Co, Bielsko-Biala, Poland; ^3^Department of Ophthalmology, Faculty of Medical Sciences in Katowice, Medical University of Silesia, Katowice, Poland; ^4^GlaucoTech Co, Katowice, Poland

**Keywords:** glaucoma–surgery, iStent, intra ocular pressure, cataracts, MIGS

## Abstract

**Purpose:**

Micro-invasive glaucoma surgery involves a group of treatment methods associated with a low rate of side effects and good effectiveness outcomes. One of the most frequently performed procedures belonging to this group is iStent microstent implantation. The aim of this study was to perform a retrospective evaluation of the safety and efficacy of a combined procedure involving cataract phacoemulsification and single iStent microstent implantation, performed simultaneously.

**Materials and methods:**

The complete medical records of 62 patients (91 eyes) were analyzed retrospectively, including the best corrected visual acuity, intraocular pressure, the mean defect of visual fields, and the number of active substances used in eye drops. The follow-up times were 1, 3, 6, 9, and 12 months after the surgical procedure.

**Results:**

A significant improvement in the best corrected visual acuity and a reduction of the intraocular pressure were achieved after the surgery. On average, after 12 months, the best corrected visual acuity improved from 0.70 (0.25) to 0.91 (0.18; *p* = 0.001), the intraocular pressure reduced from 17.76 (3.95) to 14.91 (3.04; *p* = 0.0001), and the number of active substances used in eye drops reduced from 2.07 (1.08) to 0.70 (0.06; *p* = 0001). In addition, we found that patients who initially showed higher intraocular pressure values did not benefit from surgery in the aspect of the number of active substances used in their eye drops. Intraoperative and postoperative adverse events were transient and ultimately did not affect the outcomes.

**Conclusion:**

Simultaneous cataract phacoemulsification with single iStent implantation in patients with open-angle glaucoma is a safe and effective method for reducing intraocular pressure and the number of topical medications that must be used. Having initially higher intraocular pressure values may limit the beneficial effects of iStent implantation by subordinating patients from topical treatment; thus, single iStent implantation may not be the most favorable choice in uncontrolled glaucoma cases.

## Introduction

Glaucoma refers to a heterogeneous group of diseases characterized by progressive optic neuropathy resulting from damage to retinal ganglion cells (RGCs) (1). According to estimates by the European Glaucoma Society, ~76 million people worldwide suffered from glaucoma in 2020, and forecasts indicate that, by 2040, the number of patients may increase to as many as 112 million (2). Increased intraocular pressure (IOP), age, coexisting chronic diseases (e.g., diabetes or cardiovascular diseases), high refractive errors, and, potentially, genetic background are the leading risk factors for the development of glaucoma (1, 3–5). Primary open-angle glaucoma (POAG) affects approximately 2% of the population over 40 years of age, and the percentage of patients increases with age, reaching approximately 10% in the 8th and 9th decades of life (6). POAG is the leading cause of irreversible blindness in developed countries and is a serious health and socioeconomic problem (6–13).

The current glaucoma treatment features different options depending on the type and severity of the disease (1, 14, 15). Regardless of the stage of the disease, the goal is to treat the increased IOP, which is believed to be responsible for disease progression. Among the available treatments, several types of glaucoma surgeries are available, including the gold-standard trabeculectomy and modern micro-invasive glaucoma surgery (MIGS) techniques. MIGS is a state-of-the-art glaucoma surgery that uses small incisions and specialized devices to regulate the IOP (16–22). According to the American Academy of Ophthalmology, MIGS comprises five major features: a high safety profile, minimal disruption of normal eye anatomy, an ab interno approach, efficacy—offering a meaningful IOP-lowering effect - and ease of use for patients and physicians. The introduction of MIGS techniques represented a compromise between safety and efficacy in glaucoma surgery (17, 23). Although the gold-standard penetrating bleb surgery, trabeculectomy, continues to represent the most effective surgical method for lowering the IOP, it is burdened with a greater risk of complications, including but not limited to uveal effusion, endophthalmitis, blebitis, and hypotony (24, 25). Modern glaucoma surgeries, including MIGS procedures, tend to be safer but at the cost of efficacy (26).

The iStent was specifically designed for use during cataract surgery; however, it can also be implanted as an individual procedure (27, 28). By creating a bypass, the iStent increases the aqueous humor outflow from the anterior chamber into Schlemm's canal (29). The iStent can be an effective treatment option for individuals with mild-to-moderate open-angle glaucoma who are also undergoing cataract surgery (30). It is not suitable, however, for all types and stages of glaucoma. The decision to use the iStent—or any other glaucoma treatment—depends on various factors, including the patient's specific condition, the severity of the glaucoma, and the ophthalmologist's recommendation (31).

In the present study, we aimed to evaluate the risks and benefits of parallel cataract phacoemulsification with single iStent implantation in patients with open-angle glaucoma.

## Materials and methods

### Analysis strategy

We performed a retrospective analysis of the medical records of patients who underwent the combined procedure of single iStent implantation and cataract phacoemulsification, performed simultaneously. All the procedures were performed by the same two ophthalmology specialists (IF and EK), and all the patients signed informed consent for the surgery and data usage. The approval of the local ethical committee was also obtained. We selected the records of 62 Caucasian patients−40 women and 22 men, with a mean age of 69.6 (6.8) years—for inclusion in our analysis. Among these patients, 91 eyes underwent surgical interventions−89 eyes due to POAG and 2 eyes due to secondary glaucoma associated with pseudoexfoliation syndrome. In the case of bilateral surgery, the time gap between the procedures in the right and left eye was at least 2 weeks. The indications for iStent implantation were the failure to achieve the target IOP with the maximum topical treatment, patients' adherence problems due to frequent eye drop application requirements, patients' intolerance of topical treatment, or disease progression according to the visual field test. In addition, the indication for cataract phacoemulsification was lens clouding that affected the decimal visual acuity to a level of 0.70 or lower or anisometropia/polyopia after cataract phacoemulsification in the fellow eye.

The primary inclusion criteria for surgery were as follows: being diagnosed with primary or secondary open-angle glaucoma with coexisting cataracts meeting the eligibility criteria for cataract surgery; an age of more than 18 years; a minimum best-corrected visual acuity (BCVA) of 0.10 according to the Snellen chart; an IOP in the range of 10–30 mmHg at the maximum tolerated topical treatment; and features of glaucomatous optic nerve damage in perimetry. The primary exclusion criteria for surgery were as follows: patients who underwent previous glaucoma procedures, those in whom advanced glaucoma was noted in the visual fields (according to the Hodapp–Parrish–Anderson criteria), those who showed intolerance to topical treatment, those who were pregnant or breastfeeding, those with anterior peripheral synechiae, those with corneal opacities affecting angle visibility, those with associated eye conditions, such as a history of recurrent uveitis, wet age-related macular degeneration (AMD), status post-posterior vitrectomy, or advanced diabetic retinopathy, and a lack of the patient's informed consent.

For the purpose of this analysis, we selected patients with complete and available medical records from between 2017 and 2020 who underwent the described surgeries at the Department of Ophthalmology, Medical University of Silesia, Katowice, Poland and Okulus Plus Co.

### Surgical procedures

When undergoing cataract surgery, the pupils were dilated with 1% tropicamide (Polfa, Poland); topical anesthesia was applied using 0.5% proxymetacaine hydrochloride eye drops (Alcaine, Alcon, Switzerland); and the ocular surface was rinsed with an antiseptic solution of 10% povidone-iodine. Additional intraocular anesthesia, in the form of 0.2 ml of Mydrane (Laboratories Thea, France), was additionally administered into the anterior chamber with a viscoelastic solution cover. Cataract extraction was performed via a 2.2–2.7 mm superior–temporal incision in the clear cornea, followed by the implantation of an artificial intraocular lens. For the iStent (Glaukos Corporation, San Clemente, CA, USA) implantation, the MIOSTAT solution was administered via an intracameral injection (0.1 mg/ml carbachol, Alcon, Switzerland) to constrict the pupil. The patient's head and the microscope were then rotated to enable visualization of the iridocorneal angle in the surgical gonioscope. The single iStent was inserted into Schlemm's canal, opposite the clear corneal incision. After surgery, the anti-inflammatory prophylaxis was applied by injecting cefuroxime (Aprokam, Laboratories Thea, France) into the anterior chamber.

### Follow-up strategy

The follow-up analysis assessed the BCVA, the IOP (using a Goldmann applanation tonometer), and the number of active topical substances (NAS) contained in the eye drops used by patients. The follow-up times were before the surgical procedure, followed by 1, 3, 6, 9, and 12 months after the surgery. In addition, the mean deviation (MD) of the visual fields was analyzed before and after surgery (using standard automated perimetry; Humphrey Field Analyzer 740, Zeiss, Germany, 24-2 SITA-Fast program). Intraoperative and postoperative complications were also assessed.

### Statistical analysis

For the statistical analysis, we used the IBM SPSS software (Armonk, NY, USA). Descriptive statistics are reported as the mean standard deviation (SD). The distribution of data was evaluated using the Shapiro–Wilk test. For the pairwise comparisons, we used the Wilcoxon paired-samples test. Correlations were determined by calculating non-linear Spearman correlation coefficients. A *P* < 0.05 was considered to be statistically significant. For statistical purposes, we used logarithmic visual acuity to ensure a more reliable representation of data; however, decimal visual acuity was selected for the final presentation of data in the study.

## Results

The analyzed group consisted of 62 patients (91 eyes) who underwent simultaneous cataract phacoemulsification with the implantation of an intraocular lens and a single iStent implant. The preoperative characteristics of the group are shown in Table 1. Because the analyzed patients represented a wide IOP range of 10–30 mmHg, we decided to divide them into two cohorts, depending on the preoperative IOP: subgroup A consisted of 78 eyes with preoperative IOP values of ≤ 21 mmHg, while subgroup B consisted of 13 eyes with preoperative IOP values of > 21 mmHg.

**Table 1:** Summary of mean preoperative measurements.

BCVA	0.70 (0.25)
IOP (mmHg)	17.76 (3.95)
The number of active substances in the eye drops	2.07 (1.08) 0, 1, or 2 substances: 55 eyes 3 or 4 substances: 36 eyes

### Best-corrected visual acuity

When compared to the preoperative evaluation, the BCVA improved after 1 month of follow-up by 0.21 (0.18) and 0.23 (0.16) in subgroup A and 0.14 (0.06) in subgroup B. After 3 months, it improved by 0.25 (0.15); in subgroup A, it improved by 0.24 (0.15); and in subgroup B, it improved by 0.27 (0.17). After 6 months, the BCVA improved by 0.24 (0.17); in subgroup A, it improved by 0.22 (0.03), and in subgroup B, it improved by 0.23 (0.13). After 9 months, it improved by 0.21 (0.17) 0.20 (0.16) in subgroup A, and 0.18 (0.07) in subgroup B. Finally, after 12 months, it improved by 0.21 (0.15); in subgroup A, it improved by 0.20 (0.14); and in subgroup B, it improved by 0.20 (0.15). Thus, there was a significant improvement in the BCVA at all follow-up time points for both groups (*p* < 0.05, Wilcoxon test; Table 2).

**Table 2:** Statistical analysis of BCVA evolution after the surgical procedures.

**Follow-up times [months]**	**0 vs. 1**	**0 vs. 3**	**0 vs. 6**	**0 vs. 9**	**0 vs. 12**
Overall group	0.70 (0.25) vs. 0.92 (0.16)	0.70 (0.25) vs. 0.95 (0.14)	0.70 (0.25) vs. 0.92 (0.20)	0.70 (0.25) vs. 0.90 (0.19)	0.70 (0.25) vs. 0.91 (0.18)
	*p =* 0.001	*p =* 0.001	*p =* 0.001	*p =* 0.001	*p =* 0.001
Subgroup A	0.71 (0.25) vs. 0.94 (0.14)	0.71 (0.25) vs. 0.95 (0.14)	0.71 (0.25) vs. 0.92 (0.20)	0.71 (0.25) vs. 0.91 (0.19)	0.71 (0.25) vs. 0.91 (0.19)
	*p =* 0.001	*p =* 0.01	*p =* 0.01	*p =* 0.01	*p =* 0.01
Subgroup B	0.69 (0.29) vs. 0.83 (0.25)	0.69 (0.29) vs. 0.93 (0.14)	0.69 (0.29) vs. 0.93 (0.17)	0.69 (0.29) vs. 0.85 (0.20)	0.69 (0.29) vs. 0.89 (0.18)
	*p =* 0.01	*p =* 0.001	*p =* 0.001	*p =* 0.01	*p =* 0.01

Wilcoxon paired-samples test, mean (SD).

### IOP and NAS in eye drops

After 1 month of follow-up, an overall decrease in IOP by 3.22 (2.99) mmHg was achieved, which translates into an average decrease of 18.13% compared to the preoperative IOP values; in subgroup A, it decreased by 2.51 (2.34) or 15.10%, while in subgroup B, it decreased by 6.50 (4.00) or 26.40%. Moreover, the overall NAS was reduced by 1.60 (1.14) or 77.29% compared to the preoperative values; in subgroup A, it decreased by 1.64 (1.08) or 80.39%, while in subgroup B, it decreased by 1.33 (0.50), or 59.64%. After 3 months, the IOP decreased by 3.44 (2.91) mmHg, or 19.37% [in subgroup A by 2.58 (2.23) or 15.52%; in subgroup B by 8.92 (3.00) or 36.23%] compared to the IOP before surgery. The NAS was reduced by 1.56 (1.26) or 75.36% [in subgroup A by 1.65 (1.19) or 80.88% and in subgroup B by 1.00 (0.60) or 44.84%]. After 6 months, the IOP decreased by 3.36 (2.91) mmHg, or 18.92% [in subgroup A by 2.68 (2.61) or 16.13% and in subgroup B by 9.33 (3.89) or 37.90%], and the NAS was reduced by 1.61 (1.20) or 77.78% [in subgroup A by 1.67 (1.17) or 81.86% and in subgroup B by 1.18 (1.40) or 52.92%]. After 9 months of follow-up, the IOP decreased by 3.44 (3.16) mmHg or 19.37% [in subgroup A by 2.49 (2.3)] or 14.98% and in subgroup B by 9.33 (3.89) or 37.90%], and the NAS was reduced by 1.57 (1.23) or 75.85% [in subgroup A by 1.68 (1.15) or 82.35% and in subgroup B by 0.92 (0.51) or 41.26%] compared to the values before surgery. After 12 months, the IOP decreased by 2.85 (2.40) mmHg or 16.05% [in subgroup A by 1.95 (1.68) or 11.73% and in subgroup B by 8.23 (4.64) or 33.43%], and the NAS was reduced by 1.36 (1.26) or 65.70% [in subgroup A by 1.49 (1.18) or 73.04% and in subgroup B by 0.62 (0.50) or 27.80%].

**Figure 1:**
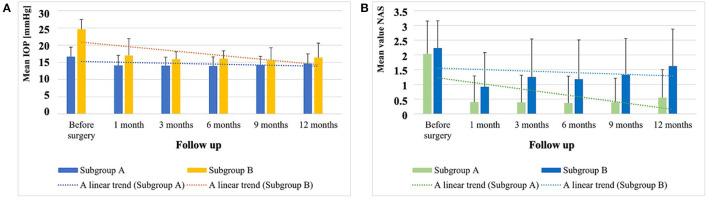
**(A)** Values of the mean IOP ± SD (mmHg) before surgery and during the following observation periods after surgery in subgroup A and subgroup B, and the linear trend of the result distribution. **(B)** NAS ± SD in eye drops before surgery and during the following observation periods after surgery in subgroup A and subgroup B, and the linear trend of the result distribution.

These results are shown in Table 3 (*p*-values were calculated using the Wilcoxon paired-samples test) and in Figure 1. In the correlation analysis, we investigated whether there is a relationship between the IOP values and the NAS before and 12 months after the procedure. In the overall group, the Spearman rank correlation coefficient before surgery was 0.04 (*p* = 0.6); 12 months after surgery, the Spearman rank correlation coefficient was 0.2 (*p* = 0.01).

**Table 3:** Statistical analysis of IOP and NAS values after the surgical procedures.

**Follow-up times [months]**		**0 vs. 1**	**0 vs. 3**	**0 vs. 6**	**0 vs. 9**	**0 vs. 12**
IOP	Overall group	17.76 (3.95) vs. 14.49 (3.39)	17.76 (3.95) vs. 14.27 (2.51)	17.76 (3.95) vs. 14.17 (2.73)	17.76 (3.95) vs. 14.36 (2.80)	17.76 (3.95) vs. 14.91 (3.04)
		*p =* 0.0001	*p =* 0.0001	*p =* 0.0001	*p =* 0.0001	*p =* 0.0001
	Subgroup A	16.62 (2.78) vs. 14.10 (2.95)	16.62 (2.78) vs. 14.01 (2.48)	16.62 (2.78) vs. 13.89 (2.69)	16.62 (2.78) vs. 14.17 (2.60)	16.62 (2.78) vs. 14.67 (2.76)
		*p =* 0.003	*p =* 0.001	*p =* 0.001	*p =* 0.001	*p =* 0.002
	Subgroup B	24.62 (2.81) vs. 17.00 (4.90)	24.62 (2.81) vs. 15.92 (2.15)	24.62 (2.81) vs. 16.09 (2.26)	24.62 (2.81) vs. 15.50 (3.73)	24.62 (2.81) vs. 16.38 (4.19)
		*p =* 0.0001	*p =* 0.0001	*p =* 0.0001	*p =* 0.0001	*p =* 0.0001
NAS	Overall group	2.07 (1.08) vs. 0.47 (0.14)	2.07 (1.08) vs. 0.51 (0.11)	2.07 (1.08) vs. 0.47 (0.10)	2.07 (1.08) vs. 0.52 (0.24)	2.07 (1.08) vs. 0.70 (0.16)
		*p =* 0.0001	*p =* 0.0001	*p =* 0.0001	*p =* 0.0001	*p =* 0.0001
	Subgroup A	2.04 (1.11) vs. 0.40 (0.19)	2.04 (1.11) vs. 0.39 (0.22)	2.04 (1.11) vs. 0.37 (0.11)	2.04 (1.11) vs. 0.39 (0.12)	2.04 (1.11) vs. 0.55 (0.25)
		*p =* 0.0001	*p =* 0.0001	*p =* 0.0001	*p =* 0.0001	*p =* 0.0001
	Subgroup B	2.23 (0.93) vs. 0.92 (0.16)	2.23 (0.93) vs. 1.25 (0.29)	2.23 (0.93) vs. 1.18 (0.33)	2.23 (0.93) vs. 1.33 (0.23)	2.23 (0.93) vs. 1.62 (0.26)
		*p =* 0.01	*p =* 0.05	*p =* 0.05	*p =* 0.06	*p =* 0.2

Wilcoxon paired-samples test, mean (SD).

### Visual fields

To assess the stage of the glaucomatous optic nerve damage, the MD of the visual fields was analyzed. In the overall group, the preoperative MD value was −4.49 (7.79), and 12 months after surgery, it was −3.27 (6.99; *p* = 0.03). In subgroup A, these values were −6.8 (7.01) and −6.4 (6.6), respectively (*p* = 0.6). In subgroup B, they were −4.94 (7.2) and −3.07 (6.8), respectively (*p* = 0.03).

### Intraoperative and postoperative complications

Among the intraoperative complications, we identified the following in our retrospective analysis: detachment of the corneal endothelium (one case) and dislocation of the implant from Schlemm's canal into the anterior chamber (one case). In terms of postoperative complications, anterior chamber bleeding was reported in three eyes. In addition, 10 eyes developed posterior capsule opacification during the follow-up period, requiring a YAG laser capsulotomy. Out of the analyzed cases, there were no cases complicated with endophthalmitis, and during the entire follow-up period, no additional glaucoma surgery was required for any of the patients.

## Discussion

The coexistence of cataracts and glaucoma is relatively common, and its prevalence increases with age (32, 33). In recent years, MIGS procedures have gained popularity, and the microstent iStent has become one of the most frequently used devices within the MIGS group (27, 29, 30, 34–39). In patients qualified for anti-glaucoma surgery who additionally show lens opacities, simultaneously combined procedures are often considered (33, 40). It is believed that cataract phacoemulsification with artificial intraocular lens implantation can be effectively and safely combined with glaucoma surgical procedures, achieving not only IOP reduction but also visual acuity improvement (33, 40).

The benefits of simultaneous cataract surgery and the implantation of an iStent have been suggested in many publications, which have also compared the results of combined procedures with those of cataract surgery alone (27, 29–32, 41). In a study conducted by Fernández-Barrientos et al. 33 eyes were randomly assigned either two iStent implants and cataract surgery (group 1) or cataract surgery alone (group 2). In group 1, the IOP and the NAS decreased significantly after 12 months of observation compared to group 2 (29). Spiegel et al. (32) conducted a 24-month multicenter study of 58 eyes after cataract phacoemulsification with the iStent implantation procedure; they reported the procedure to be safe and effective in reducing the IOP and NAS in eye drops. Arriola-Villalobos et al. (31) compared the results of 19 eyes with concomitant open-angle glaucoma (including pseudoexfoliative and pigmentary glaucoma) and cataracts that underwent phacoemulsification and the implantation of an intraocular lens, along with the implantation of a single iStent implant. The 3-year follow-up visit showed that this treatment method was both safe and effective.

Tan et al. (27) assessed the safety and efficacy of cataract phacoemulsification combined with single iStent implantation in open-angle glaucoma over a 3-year follow-up period. Forty-one eyes were examined, of which thirty-six completed the 3-year follow-up. According to the study report, the combined treatment turned out to be both safe and effective. Fea et al. (30) presented the results of the observation of 36 eyes with cataracts and POAG who were randomly assigned to cataract surgery combined with iStent implantation or phacoemulsification alone. The authors showed that both methods reduced the NAS in the drops used by patients. However, the efficacy of cataract phacoemulsification alone in reducing the IOP faded over time, while after the combined procedure, the lower IOP values remained constant over the observation period. In our study, the group of patients who underwent simultaneous cataract surgery and single iStent implantation showed an improvement in BCVA that was observed at all follow-up points, with the values being 30% better after 12 months compared to before the surgery. However, the BCVA improvement was mostly due to the cataract phacoemulsification rather than the implantation of the iStent implant itself. Tan et al. (27) included 41 patients in their study, implanting a single iStent in conjunction with cataract surgery. The authors observed an improvement in the postoperative BCVA by more than two lines as early as 1 month after the procedure. This value remained at a similar level for the next few months of observation. Katz et al. (35) carried out an 18-month follow-up study of 119 patients with POAG who had one, two, or three iStent implants. One group featured cases of pseudophakia (i.e., the single-stent group), and the other groups included eyes with their own lens. The postoperative BCVA values did not differ significantly compared to the preoperative ones. In the literature, one can find a comparison of the effectiveness of the implantation of an iStent alone or with simultaneous cataract surgery. In some studies, the results suggest that reducing the IOP in patients with POAG is sufficient using iStent implantation alone (42), while other researchers have found greater benefits when the two procedures are combined (19, 43–45). When comparing cataract surgery alone with glaucoma surgery, studies have shown that the second method gives better results (35). Another study showed that the implantation of two or three iStents gives even better results than a single implant (40).

We observed that, in the case of simultaneous iStent implantation and cataract surgery, after 12 months of observation, the IOP decreased by an average of 2.85 ± 4.40 mmHg (16.05%) to the level of 14.91 ± 3.04 mmHg, with an average NAS in eye drops of 0.70 ± 1.06 (i.e., a decrease by 1.36 ± 1.26). Moreover, after 12 months of observation in the overall group, the NAS used in eye drops—compared to the values before the surgery—decreased in 68 eyes (74.73%), while 54 eyes (59.34%) did not require the use of eye drops at all. For 20 eyes, the NAS remained at the same level, while for 3 eyes, it was necessary to increase the NAS. It should be noted that no additional glaucoma surgery was required for any of the patients during the entire 12-month follow-up period.

Our results are comparable to those presented in the study by Spiegel et al. (32), who reported that the IOP decreased by an average of 4.3 mmHg and the NAS by 1.2 after 12 months of observation. In contrast, our results were more favorable than those in the study by Fea et al. (21), who reported that the IOP decreased by an average of 1.7 mmHg and the NAS by 0.4 after 12 months of observation. Finally, our results were worse than those in the study by Tan et al. (27), who reported that the IOP decreased by an average of 5.3 mmHg and the NAS by 1.6 after 12 months of observation. The differences between the results may arise from the different group sizes and baseline values used to qualify patients for the procedure.

The first-line treatment for glaucoma is usually IOP-lowering drops (14, 15). Considering that a large proportion of patients diagnosed with glaucoma are elderly people with many systemic diseases, it can be assumed that their adherence to the rules of using eye drops is often less than ideal. Non-compliance, in turn, may lead to disease progression and the gradual loss of vision, which translates into the disability of patients and high costs for health and social care (46). Based on our data and the analysis of the literature, we have shown that the reduced number of topical medications required after a single iStent implantation makes this type of surgery a good option for patients who are intolerant to eye drops and/or who have difficulties using them. Katz et al. (47) also suggested that if a greater reduction in the IOP is required, the implantation of more than one iStent may be considered.

There is strong evidence suggesting that IOP fluctuates daily when using eye drops and that their effect on IOP regulation may be limited (48, 49). In the analyzed group of patients before the procedure, the NAS did not correlate with the IOP values. It can therefore be concluded that topical medications did not achieve the target IOP. For this reason, the patients were referred for surgical treatment. After concurrent cataract surgery with single iStent implantation, the correlation between the NAS and IOP was restored, thus restoring the equilibrium state and achieving therapeutic success. In our study, we subtracted the uncontrolled glaucoma patients (subgroup B) from the overall study group to investigate whether higher initial IOP values would limit the effectiveness of single iStent implantation. Despite the effective lowering of the IOP 12 months after surgery, these patients did not benefit from the surgery in terms of the NAS used in eye drops, as this value was similar 12 months after surgery to that reported preoperatively. This may suggest that uncontrolled glaucoma patients with higher initial IOP values may not be the best candidates for single iStent implantation as they may require more radical surgery or the implantation of multiple iStents. Indeed, some studies have shown the triple implantation of the iStent infinite implant to be effective in the treatment of uncontrolled glaucoma cases (50). The implantation of the iStent not only reduces the IOP but also theoretically limits daily IOP fluctuations, thus minimizing the risk of glaucoma progression. We did not observe changes between the preoperative and postoperative visual field test results. However, a significant improvement in the visual field MD was obtained after 12 months compared with the results before the surgery, which could be explained by the following: First, we are aware that a follow-up period of 1 year could be too short to observe visual field changes. Second, the MD of visual fields considers the diffuse defects, which could be caused by the cataract itself. Finally, it is known that if the damaging factor is eliminated in the early stages of the disease—that is, the increased IOP—the function of the RGCs may improve; this is reflected in our analysis, especially in subgroup B, in which there were higher initial IOP values (51–53). The Advanced Glaucoma Intervention Study (AGIS) (54) also confirmed the relationship between low IOP and reduced glaucoma progression risk in patients with POAG. Myers et al. (55) included in their 4-year follow-up 80 patients (80 eyes) with open-angle glaucoma resistant to topical treatment. Each patient had a history of trabeculectomy and the subsequent implantation of two iStent implants and one iStent Supra implant. As a postoperative topical treatment, travoprost eye drops were recommended. None of the patients required additional anti-glaucoma surgery throughout the 4-year follow-up period. The applied combination treatment achieved IOP control, and the postoperative visual field results remained stable throughout the observation period.

The various intraoperative and postoperative complications that have occurred in patients have been reported in the literature. The most frequently mentioned complication was the presence of blood in the anterior chamber (36, 40). In our study, we found reports of three patients with this complication. However, it is known that some blood reflux through the iStent is often observed after implant placement, which indicates the correct placement of the implant in Schlemm's canal (44). In a study by Tan et al. (27), after cataract surgery with iStent implantation, blood cells were observed in the anterior chamber of the eye in one patient (2.44% of examined eyes). This symptom resolved within 1 week. Similarly, in a study by Buchacra et al. (41), among 10 examined eyes, there were three cases of blood reflux into the anterior chamber during the placement of the iStent implant in Schlemm's canal. Fernández-Barrientos et al. (29) described slight blood reflux from the implant as a positive sign, indicating the correct placement of the iStent in Schlemm's canal.

The analyzed literature also described difficulty inserting the implant into Schlemm's canal as a possible intraoperative complication (55). In this study, similar problems were described during surgery. In one case, implant dislocation from Schlemm's canal into the anterior chamber occurred, while in another case, detachment of the corneal endothelium occurred, caused by mechanical damage from the iStent guide.

There are several limitations of our study. The size of the study group and subgroups does not fully represent the glaucoma population. In addition, the retrospective construction of the study limited the analysis to the available medical records and did allow us to enrich the analysis with some more sensitive markers, such as the visual field pattern standard deviation (PSD), electrophysiology, or corneal endothelial cell count.

## Conclusion

Simultaneous cataract phacoemulsification and single iStent implantation in patients with open-angle glaucoma is a safe and effective method to reduce the IOP and the number of topical medications used and to prevent visual field deterioration. Initially, higher IOP values may limit the beneficial effects of iStent implantation because these patients continue to require topical treatment after the surgery; thus, a single iStent implantation may not be the most favorable choice in uncontrolled glaucoma cases. However, simultaneous cataract phacoemulsification and single iStent implantation in patients with uncontrolled open-angle glaucoma may help to restore the equilibrium state between the IOP and topical medications, resulting in therapeutic success.

## Data availability statement

The original contributions presented in the study are included in the article/supplementary material, further inquiries can be directed to the corresponding authors.

## Ethics statement

The studies involving humans were approved by the Ethical Committee of Medical University of Silesia. The studies were conducted in accordance with the local legislation and institutional requirements. The participants provided their written informed consent to participate in this study.

## Author contributions

MH-S: Data curation, Investigation, Methodology, Writing—original draft. AS: Formal analysis, Methodology, Supervision, Validation, Writing—review & editing. IF: Investigation, Methodology, Writing—review & editing. EM-K: Conceptualization, Data curation, Funding acquisition, Writing—review & editing.
